# PRL-1 overexpressed placenta-derived mesenchymal stem cells suppress adipogenesis in Graves’ ophthalmopathy through SREBP2/HMGCR pathway

**DOI:** 10.1186/s13287-021-02337-2

**Published:** 2021-05-29

**Authors:** Mira Park, Jae Yeon Kim, Jun Mo Kang, Hey Jin Lee, Jasvinder Paul Banga, Gi Jin Kim, Helen Lew

**Affiliations:** 1 Department of Ophthalmology, Bundang CHA Medical Center, CHA University, Seongnam, Gyeonggi-do 13496 Republic of Korea; 2 Department of Biomedical Science, CHA University, Seongnam, Gyeonggi-do 13488 Republic of Korea; 3 Research Institute of Placental Science, CHA University, Seongnam, Gyeonggi-do 13488 Republic of Korea; 4 CHA Advanced Research Institute, CHA University, Seongnam, Gyeonggi-do 13488 Republic of Korea; 5 Faculty of Life Sciences & Medicine, King’s College London, London, SE5 9NU UK

**Keywords:** Graves’ ophthalmopathy, Graves’ disease, GO animal model, hPMSCs, Thyroid disease, adipogenesis

## Abstract

**Background:**

Graves’ ophthalmopathy (GO) is a disorder, in which orbital connective tissues get in inflammation and increase in volume. Stimulants such as thyroid-stimulating hormone (TSH), insulin-like growth factor 1(IGF-1), IL-1, interferon γ, and platelet-derived growth factor cause differentiation into adipocytes of orbital fibroblasts (OFs) in the orbital fat and extraocular muscles. Human placental mesenchymal stem cells (hPMSCs) are known to have immune modulation effects on disease pathogenesis. Some reports suggest that hPMSCs can elicit therapeutic effects, but to date, research on this has been insufficient. In this study, we constructed PRL-1 overexpressed hPMSCs (hPMSCs^PRL-1^) in an attempt to enhance the suppressive function of adipogenesis in GO animal models.

**Methods:**

In order to investigate the anti-adipogenic effects, primary OFs were incubated with differentiation medium for 10 days. After co-culturing with hPMSCs^PRL-1^, the characteristics of the OFs were analyzed using Nile red stain and quantitative real-time polymerase chain reaction. We then examined the in vivo regulatory effectiveness of hPMSCs^PRL-1^ in a GO mouse model that immunized by leg muscle electroporation of pTriEx1.1Neo-hTSHR A-subunit plasmid. Human PMSCs^PRL-1^ injection was performed in left orbit. We also analyzed the anti-adipogenic effects of hPMSCs^PRL-1^ in the GO model.

**Results:**

We found that hPMSCs^PRL-1^ inhibited adipogenic activation factors, specifically *PPARγ*, *C/EBPα*, *FABP4*, *SREBP2*, and *HMGCR*, by 75.1%, 50%, 79.6%, 81.8%, and 87%, respectively, compared with naïve hPMSCs in adipogenesis-induced primary OFs from GO. Moreover, hPMSCs^PRL-1^ more effectively inhibited adipogenic factors *ADIPONECTIN* and *HMGCR* by 53.2% and 31.7%, respectively, than hPMSCs, compared with 15.8% and 29.8% using steroids in the orbital fat of the GO animal model.

**Conclusion:**

Our findings suggest that hPMSCs^PRL-1^ would restore inflammation and adipogenesis of GO model and demonstrate that they could be applied as a novel treatment for GO patients.

**Supplementary Information:**

The online version contains supplementary material available at 10.1186/s13287-021-02337-2.

## Introduction

Graves’ disease (GD) is an antibody-mediated autoimmune disease associated with thyroid-stimulating hormone receptor (TSHR) in the thyroid gland that causes hyperthyroidism [1]. Graves’ ophthalmopathy (GO) is an autoimmune inflammatory disease in which TSHR-stimulated antibodies and TSHR affect cells in surrounding tissues [2]. Thyroid eye symptoms include proptosis, eyelid retraction, exposure keratopathy, strabismus, limitation of eye movement, compressive optic neuropathy, and disfigured appearance. T cell infiltration and accumulation of fibroblastic glycosaminoglycan are the major pathologic findings of autoantibody-involved inflammatory and fibrotic reactions in retro-orbital fat and extraocular muscles (EOMs). Edematous swelling of the EOMs and adipogenesis result in an increase in the volume in the bony orbital cone; as such, typical GO symptoms contain irritation, tearing, orbital pain, vision loss, diplopia, corneal ulceration, and even blindness [3].

Human placental mesenchymal stem cells (hPMSCs) offer multilineage differentiation potential and especially strong immunomodulatory abilities for regenerative medicine applications [4]. Compared with other MSCs, PMSCs have a superior immunomodulatory advantage, resulting in highly expressed human leukocyte antigen (HLA)-ABC and HLA-G [5]. Furthermore, we previously generated phosphatase of regenerating liver-1 (PRL-1) overexpressed hPMSCs and demonstrated that CD13, CD90, CD105, and MHC class I including HLA-ABC and HLA-G were positive, whereas MHC class II including HLA-DR was negative [6]. Hence, the therapeutic effects of hPMSCs are considered with immunosuppression-mediated replacement of damaged tissues.

PRL-1 (protein tyrosine phosphatase type IVA member 1; PTP4A1; PTPCAAX1) is a member of prenylated PTPs and was initially identified as an immediate-early gene for hepatic regeneration [7]. Its gene enhances cell proliferation during protein prenylation for post-translational lipid modification thorough the mevalonate metabolic pathway. The key enzyme 3-hydroxy-3-methylglutaryl coenzyme A (HMG-CoA) reductase is modulated through AMP-activated protein kinase (AMPK) [8]. In addition, PRL-1 expression involved in the oxidative status and glutathione (GSH) system in mammalian retina and photoreceptors [9].

In this study, we compared our results with those of conventional treatments in an experimental mouse model of GO [10, 11]. In addition, we demonstrated the immunomodulatory effects of functionally enhanced hPMSCs in the GO models.

## Materials and methods

### Cell culture of hPMSCs and hPMSCs^PRL-1^

OFs from patients with GO were collected as described previously [12]. The protocol for OF preparation was approved by the Institutional Review Board of Bundang CHA Medical Center, Seongnam, Republic of Korea (IRB-2018-01-007), and consent was obtained from all patients. Orbital adipose tissue was obtained from GO patients during orbital fat decompression and control individuals without GO history during blepharoplasty. Tissues were chopped and incubated with collagenase (0.25 mg/mL; Thermo Fisher Scientific, Waltham, MA, USA) for 1 h at 37 °C in a shaking incubator. After digestion, the tissues were placed directly in culture dishes with DMEM/F12 containing 20% fetal bovine serum (FBS; Thermo Fisher Scientific) and 1% penicillin/streptomycin (Thermo Fisher Scientific). The ranges from fifth to eighth cell passages were used in the experiments. Human PMSCs were collected as described previously [13]; the associated protocol was approved by the Institutional Review Board of Gangnam Medical Center, Seoul, Republic of Korea (IRB-07-18). All patients consented to the proper use for research. hPMSCs were cultured in α-modified minimal essential medium (α-MEM; HyClone, Logan, UT, USA) supplemented with 10% FBS (Gibco), 1% P/S (Gibco), 1 μg/mL heparin (Sigma-Aldrich, St. Louis, MO, USA), and 25 ng/mL human fibroblast growth factor-4 (hFGF-4; Peprotech, Rocky Hill, NJ, USA). PRL-1 plasmid purchased from Origene (#RG200435; Rockville, MD, USA) and hPMSCs^PRL-1^ were established using The P1 Primary Cell 4D nucleofector system [14]. Normal, OFs with GO patients, hPMSCs, and hPMSCs^PRL-1^ were maintained at 37 °C in a humidified atmosphere containing 5% CO_2_.

### Adipocyte differentiation and Nile red staining

Normal and GO-derived OFs (5 × 10^3^/cm^2^) were cultured in a six-well plate and incubated in serum-free DMEM/F12 supplemented with 33 μM biotin, 17 μM pantothenic acid, 0.2 nM triiodothyronine (T_3_), 10 μg/mL transferrin, 0.2 μM carbaprostacyclin (cPGI_2_; Cayman Chemical, Ann Arbor, MI, USA), 0.1 mM isobutylmethylxanthine (IBMX), 1 μM dexamethasone, and 1 μM insulin (Sigma-Aldrich). The differentiation-induced medium was replaced every day for 4 days. The medium exchanged to a maturation medium without 1 μM dexamethasone and 0.1 mM IBMX (all from Sigma-Aldrich) for 6 days and was replaced every other day. Lipid droplets were stained using 0.5 μg/mL Nile red solution (Sigma-Aldrich).

### Co-culture experiments

After adipocyte differentiation, normal and GO-derived OFs were co-cultured with hPMSCs and hPMSCs^PRL-1^ (5 × 10^3^/cm^2^) in upper Transwell inserts (8-μm; Corning, NY, USA) in α-MEM (HyClone) supplemented with 1% P/S (Gibco) for 24 h at 37 °C in a humidified atmosphere containing 5% CO_2_.

### Quantitative real-time polymerase chain reaction

Total RNA was isolated from orbital tissues of the GO model and human OFs using TRIzol reagent (Ambion, Carlsbad, CA, USA). Quantitative real-time polymerase chain reaction (qRT-PCR) was performed with IQ SYBR Green Supermix (Bio-Rad Laboratories, Hercules, CA, USA). Gene expression was quantified by the delta CT method, and real-time PCR reactions were performed using a CFX-96 system (Bio-Rad Laboratories). Tables 1 and 2 list the nucleotide sequences of all primers used.

**Table 1:** Human primer sequences using quantitative real time polymerase chain reaction

Genes		Primer sequences	Tm
Adipsin	Forward	5′-GGGCAGCGTGTACTTATCCT-3′	55
	Reverse	5′- AGAACCCCAAGATGCACAAC -3′	
PPAR-γ	Forward	5′- GCGGCTACTACAACCAGAGC -3′	55
	Reverse	5′- GCACATGGCACGTGTATCTC -3′	
Adiponectin	Forward	5′-GAGCTGACGTGGAAGATGAG-3′	55
	Reverse	5′-CTTCAAGTGCTGTCTGATTCCAATG-3′	
Leptin	Forward	5′-ATGCTGCAAACTGACCACGC-3′	55
	Reverse	5′-GCTTCGCTTTGCCAATGCTT-3′	
LPL	Forward	5′-TGAGTTTGCAGAAGTTTCCA-3′	60
	Reverse	5′-CCTTTGCCTCAGCATAGTTT-3′	
FABP4	Forward	5′-GCATGGCCAAACCTAACATGA-3′	55
	Reverse	5′-CCTGGCCCAGTATGAAGGAAA-3′	
C/EBPα	Forward	5′-TGTATACCCCTGGTGGGAGA-3′	60
	Reverse	5′-TCATAACTCCGGTCCCTCTG-3′	
C/EBPβ	Forward	5′-CTTCAGCCCGTACCTGGAG-3′	60
	Reverse	5′-GGAGAAGGAAGTCGTGGTGC-3′	
TSHR	Forward	5′-GACACTGAAGCTGTACAACAATGG-3′	60
	Reverse	5′-AGACACGTCCAGCAAGCTTGGT-3′	
SREBP2	Forward	5′-CAAGGCCCTGGAAGTGACAGA-3′	60
	Reverse	5′-AGGAACTCTGCTGCCCATCTG-3′	
HMGCR	Forward	5′-GCCTGGCTCGAAACATCTGAA-3′	60
	Reverse	5′-CTGACCTGGACTGGAAACGGATA-3′	
ICAM-1	Forward	5′-CAGTCACCTATGGCAACGACTC-3′	60
	Reverse	5′-CTCTGGCTTCGTCAGAATCAC-3′	
IL-1β	Forward	5′-ATGAGTGCTCCTTCCAGGA-3′	60
	Reverse	5′-GATAGGTTCTTCAAAGATG-3′	
TNF-α	Forward	5′-CCAGAGGGAAGAGTTCCCCA-3′	55
	Reverse	5′-TCAGCTTGAGGGTTTGCTACAAC-3′	
IL-6	Forward	5′-TCCACAAGCGCCTTCGGTCCAGTTG-3′	55
	Reverse	5′-AGAGGTGAGTGGCTGTCTGTGTGGG-3′	
TGF-β1	Forward	5′-TACCAGAAATACAGCAACAATTCC-3′	55
	Reverse	5′-AAAGCCCTCAATTTCCCCTCC-3′	
TGF-β2	Forward	5′-TGGTGAAAGCAGAGTTCAGAG-3′	55
	Reverse	5′-CACAACTTTGCTGTCGATGTAG-3′	
GAPDH	Forward	5′-TCCTTCTGCATCCTGTCAGCA-3′	60
	Reverse	5′-CAGGAGATGGCCACTGCCGCA-3′

**Table 2:** Mouse primer sequences using quantitative real time polymerase chain reaction

Genes		Primer sequences	Tm
C/ebpα	Forward	5′-CGCAAGAGCCGAGATAAAGC-3′	60
	Reverse	5′-CGGTCATTGTCACTGGTCAACT-3′	
Leptin	Forward	5′-GACACCAAAACCCTCATCAAGAC-3′	60
	Reverse	5′-CGTGTGTGAAATGTCATTGATCCT-3′	
Adiponectin	Forward	5′-GGAACTTGTGCAGGTTGGAT-3′	55
	Reverse	5′-CCTTCAGCTCCTGTCATTCC-3′	
Fabp4	Forward	5′-TCGATGAAATCACCGCAGAC-3′	50
	Reverse	5′-TGTGGTCGACTTTCCATCCC-3′	
Hmgcr	Forward	5′-CACCTCTCCGTGGGTTAAAA-3′	60
	Reverse	5′-GAAGAAGTAGGCCCCCAATC-3′	
Icam-1	Forward	5′-AACAGAATGGTAGACAGCAT-3′	60
	Reverse	5′-TCCACCGAGTCCTCTTAG-3′	
Il-1β	Forward	5′-GCCACCTTTTGACAGTGATGAG-3′	55
	Reverse	5′-CCTGAAGCTCTTGTTGATGTGC-3′	
Il-6	Forward	5′-TCTATACCACTTCACAAGTCGGA-3′	60
	Reverse	5′-GAAT TGCCATTGCACAACTCTTT-3′	
Tnf-α	Forward	5′-GTCTACTGAACTTCGGGGTGA-3′	60
	Reverse	5′-CTCCTCCACTTGGTGGTTTG-3′	
Tgf-β2	Forward	5′-TCGACATGGATCAGTTTATGCGCA-3′	60
	Reverse	5′-CCCTGGTACTGTTGTAGATGGA-3′	

### Development of an experimental mouse model of GO using female BALB/c mice

We produced a GO mouse model using immunization by leg muscle electroporation of pTriEx1.1Neo-hTSHR A-subunit plasmid, as described previously [11]. Establishment of the GO model is described in an earlier report [12].

### Immunomodulation

**Fig. 1:**
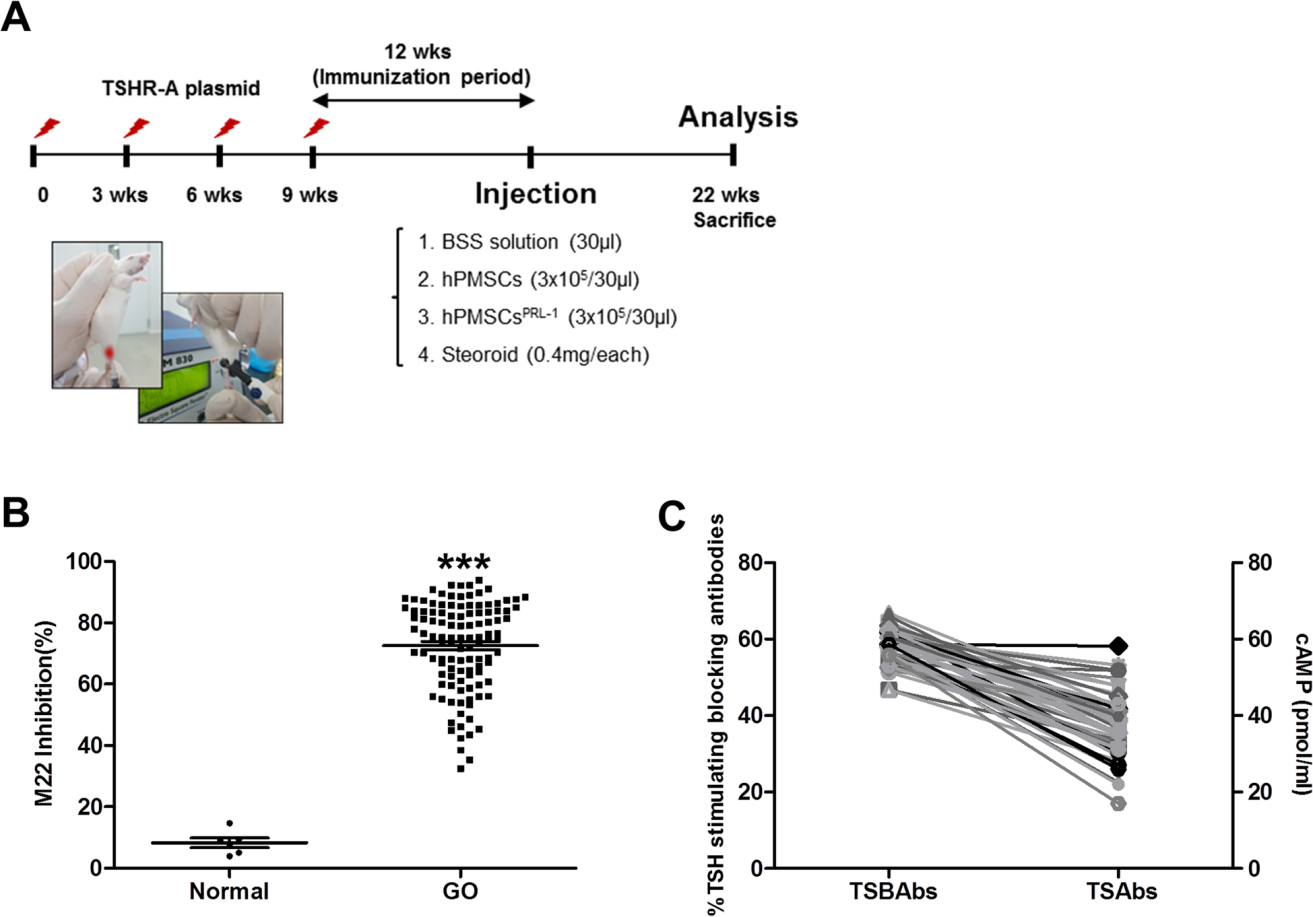
Histologic analysis of animals undergoing experimental GO treated with hPMSC and hPMSCs^PRL-1^. **a** The scheme of in vivo experiment for construction of GO animal model and stem cell injection. **b** Anti-TSHR Ab inhibition. **c** TSBAbs and TSAbs in serum of disease mice at 12 weeks after last immunization with pTriEx-1.1 TSH receptor (TSHR) A-subunit plasmid in muscle combined with electroporation. Significantly different values between groups are indicated with asterisk (***p < 0.0001), normal *n* = 6, GO *n* = 113

At 21 weeks after the first immunization (Fig. 1a), all immune animals were bled to assess induced anti-TSHR antibody level. The GO animals were divided into the following groups: a treatment group injected with hPMSCs (3 × 10^5^ cells/30 μL), a treatment group injected with hPMSCs^PRL-1^ (3 × 10^5^ cells/30 μL), a treatment group injected with steroids (0.4 mg/each, triamcinolone acetonide, Dongkwang Pharmaceutical Co., Hanmi, South Korea), and a sham group (30 μL BSS PLUS). Intra-orbital injection was performed on the left orbit. After single hPMSC injection, the animals were sacrificed after 1 week, after which their blood was collected for serum, and orbital tissue was excised for histopathological analyses.

### Measurement of antibodies to TSHR

We confirmed that GO animal models were achieved prior to dividing the mice into treatment groups, based on serum analysis at 21 weeks using the anti-TSH receptor antibody (TRAb) from a fast enzyme-linked immunosorbent assay (ELISA) commercial kit (EUROIMMUN, Luebeck, Germany); experiments were carried out according to the manufacturer’s instructions. The result is expressed as a percentage suppression of the M22 binding to the immobilized TSHR in the plate wells. The activity levels of thyroid-stimulating antibodies (TSAb) and TSH-stimulating blocking antibodies (TSBAb) in the serum were analyzed in TSHR expressing Chinese hamster ovary (CHO) cells, as described in a previous study [15].

### Orbital tissue histopathology

Quantification of the adipose tissue around the optic nerve was performed using ZEISS ZEN Microscope Software (Carl Zeiss, Jena, Germany). Adipose tissue area was measured as the difference between the entire adipose area and that of the axon area of the eye tissue. The cross-sectional area of the orbital fat was normalized to the region of the other side adipose tissue area of each mouse. The area of adipose tissues in the orbital sections of each mouse was evaluated in all groups.

### Measurement of the cross-sectional area of the optic nerve, lacrimal gland, and extraocular muscles

Quantification of the cross-sectional area of the orbital structure, including the optic nerve, lacrimal gland, and EOMs, was calculated using the formula: [{(total length × (total breadth)}/2]. The cross-sectional area of the oculus sinister was normalized with respect to the oculus dexter.

### Immunoblot analyses of target proteins and cell signaling pathways

Lysates were gained from orbital tissues, which were injected with BSS (sham), hPMSCs, hPMSCs^PRL-1^, or steroids by homogenization with PRO-PREP solution (Intron, Gyeonggido, Korea). Equal concentration of protein were loaded by sodium dodecyl sulfate-polyacrylamide gel electrophoresis and transferred to membranes. The membranes were incubated with anti-ICAM-1 (Thermo Fisher Scientific), TGFβ2 (GeneTex, Irvine, CA, USA), TSHR (NSJ Bioreagents, San Diego, CA, USA), or α-tubulin (GeneTex). After washing, it was incubated at room temperature for 2 h with horseradish peroxidase-conjugated anti-rabbit or mouse IgG secondary antibodies at a dilution of 1:5000 (GeneTex). The immune response bands were visualized with enhanced chemiluminescence solution (Bio-Rad Laboratories) and detected using an ImageQuant LAS 4000 (GE Healthcare Life Sciences, Little Chalfont, UK).

### Statistical analyses

Data analysis was performed using GraphPad Prism (GraphPad Software, La Jolla, CA, USA). Statistically significant differences were identified using *t* test or nonparametric statistical test, followed by a Mann–Whitney *U* test at a significance level of 5%.

## Results

### Characterization studies

To explore the treatment abilities of hPMSCs, we produced a GO animal model, as described in a recent study [10]. Using electroporation, hTSHR A-subunit plasmid was injected to mice (Fig. 1a). After an immunization period, we collected serum from the GO model to evaluate for antibodies to TSHR. In the analysis of TSH-binding inhibitory immunoglobulin (TBII) assay with anti-TSH receptor (TRAb) ELISA, the GO models showed an inhibition of more than 72% of the labeled TSH binding activity compared with the control group (Fig. 1b). Measurement of anti-TSHR antibody subtypes in the GO model indicated animals were positive for TSAbs and TSBAbs (Fig. 1c). TSAbs values ranged from a minimum of 17 to a maximum of 58 pmol/mL, and TSBAbs were found to have 46–66% TSH-stimulating blocking abilities (Fig. 1c). TSBAbs values were higher than TSAbs in the GO animal model (Fig. 1c).

### Pathology assessment

**Fig. 2:**
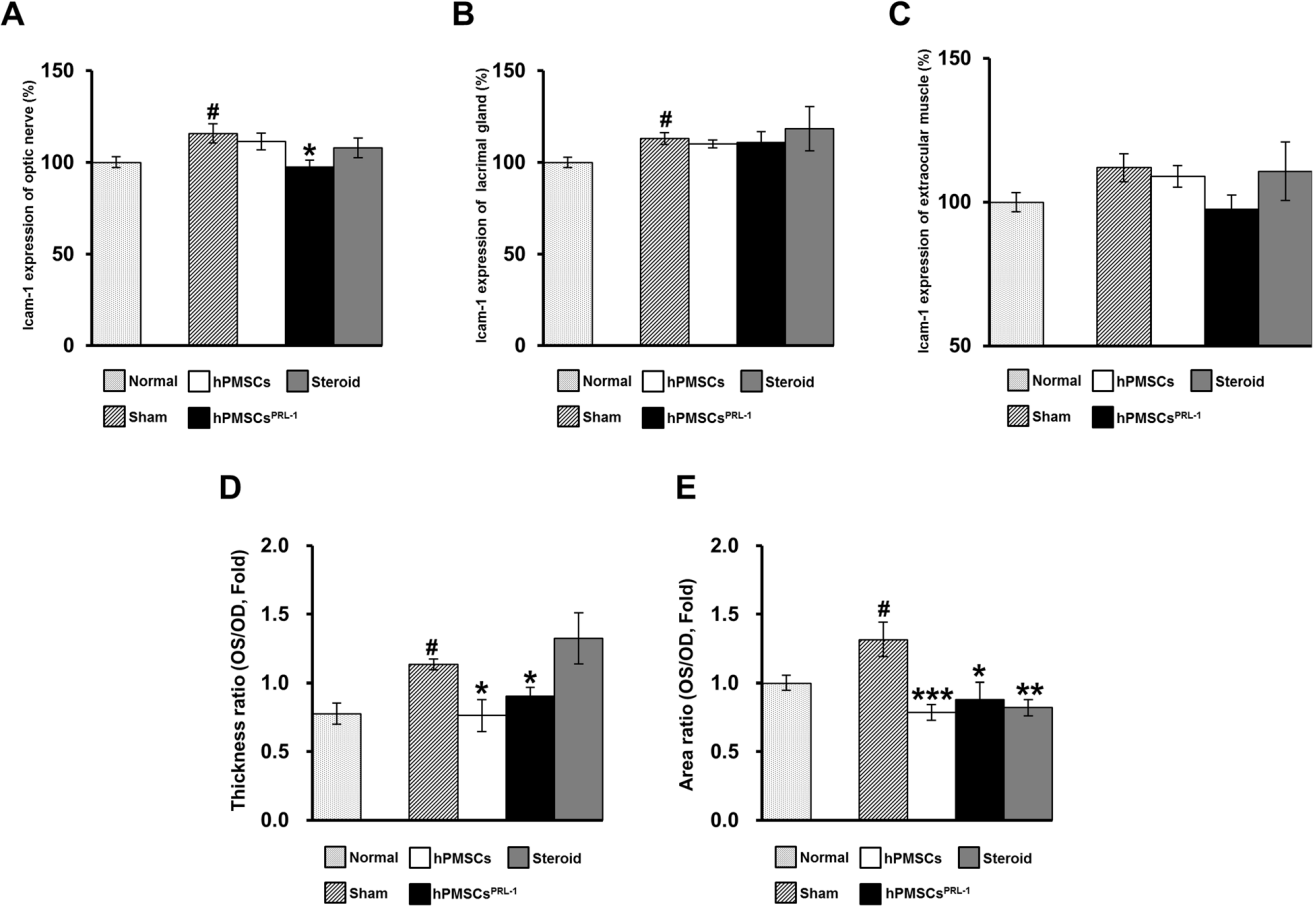
Histologic analysis of GO animals treated with hPMSCs^PRL-1^ or steroid injection. ICAM-1 staining of orbital tissue, by orientating the paraffin block for sectioning with optic nerve as an anatomical landmark. ICAM-1 expressions were measured in **a** optic nerve, **b** lacrimal gland, and **c** extraocular muscle tissue of retrobulbar. The **d** thickness and **e** expansion of retrobulbar adipose tissue around the optic nerve were measured. Data was presented as the fold changes (means ± SEM) of thickness and adipose volume around optic nerve compared with the sham of each group. Significantly different values between groups are indicated with mark (*p < 0.05, **p < 0.01, ***p < 0.001 vs sham; #*p* < 0.05 vs normal). **a**–**d** Normal *n* = 6, GO *n* = 4; **e** *n* = 6/each group

We evaluated the pathology of GO groups and analyzed Icam-1 protein values in the eyes of GO animal models. In Sham, Icam-1 expression was significantly increased compared with that of a normal optic nerve (Fig. 2a). Additionally, increased Icam-1 was significantly reduced by hPMSCs^PRL-1^ (Fig. 2a). Regulation of Icam-1 expression by hPMSC injection was not evident in lacrimal gland and EOM tissues (Fig. 2b, c). We measured the volume of orbital tissue in each group using hematoxylin and eosin staining. The optic nerve thickness of the sham group was significantly increased compared with that of normal mice (Fig. 2d). Human PMSCs and hPMSCs^PRL-1^ were found to reduce the thickness of the optic nerve in the GO model, but steroid injection did not have the same effect (Fig. 2d). At 1 week post-transplantation, the retrobulbar adipose tissues of hPMSC, hPMSCs^PRL-1^, and steroid-injected mice were significantly reduced in terms of pathologic expansion (Fig. 2e).

### Enhanced human PMSC transplantation decrease adipogenesis in the GO animal model

**Fig. 3:**
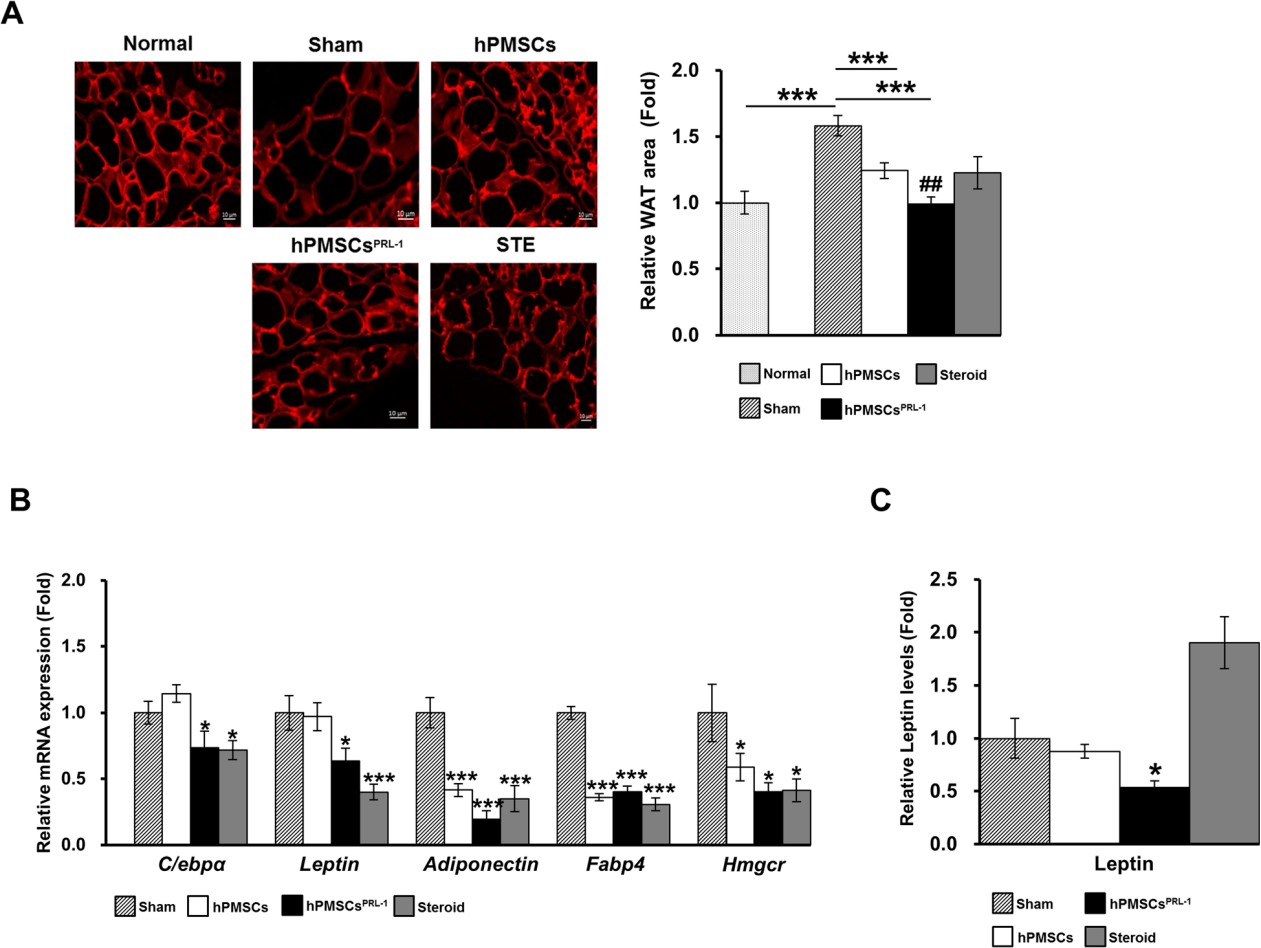
hPMSCs^PRL-1^ inhibit adipogenesis in GO animal models. **a** Quantification of white adipose tissues area stained with perilipin, an adipocyte marker, was measured around optic nerve of GO models at 1 week post-transplantation (*n* = 3/each group). **b** Adipogenesis-related genes such as *C/ebpα*, *leptin*, *adiponectin*, and *Fabp4* mRNA were determined (*n* = 6/each group). **c** Leptin levels in serum of GO models were measured (*n* = 6/each group). Data was presented as the fold changes (means ± SEM). Significantly different values between groups are indicated with asterisk (*p < 0.05, ***p < 0.001 vs age-matched sham; ##*p* < 0.05 vs hPMSCs)

We investigated expression changes in adipogenic-related target genes in orbital tissue from the GO model. After staining adipose cells with perilipin, an adipocyte marker protein, the white adipose tissue (WAT) cell area around the optic nerve was measured (Fig. 3a). The WAT area was significantly larger by 58.3% than the normal tissue area. Additionally, both hPMSC and hPMSC^PRL-1^ injections significantly inhibited adipose cell area, in contrast to the steroid injection (Fig. 3a). With regard to changes in adipogenic gene transcriptions such as *adiponectin*, *fatty acid-binding protein 4* (*Fabp4*), and *Hmgcr* exhibited significant reductions by all therapeutic treatments (Fig. 3b). For *C/ebpα* and *Leptin*, hPMSCs^PRL-1^ and steroid treatments significantly reduced mRNA expression (Fig. 3b). Additionally, hPMSCs^PRL-1^ reduced the leptin levels in the GO animal model (Fig. 3c). We also investigated changes in the expressions of inflammatory-related factors together with adipogenesis factors. Our results indicate that all therapeutic candidates have the ability to inhibit mRNA expressions of pro-inflammatory factors (Supplementary Fig. S1A). Additionally, the results revealed that hPMSCs, hPMSCs^PRL-1^, and steroid post-transplantation significantly inhibited Icam-1, Tgfβ2, and Tshr protein expressions (Supplementary Fig. S1B).

### Enhanced hPMSC co-culture inhibits adipogenesis in GO-derived OFs

**Fig. 4:**
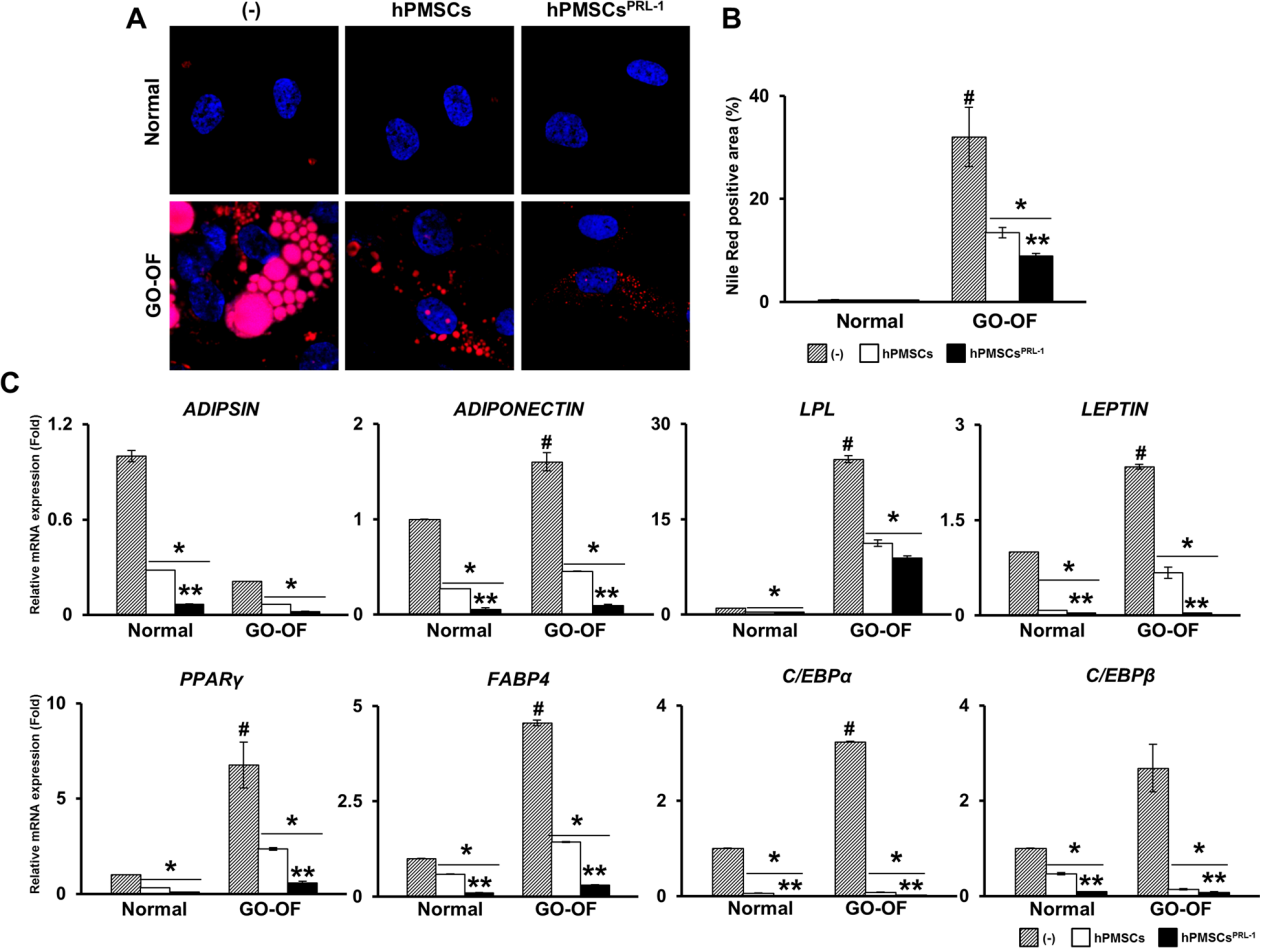
hPMSCs ^PRL-1^ co-culture inhibit adipogenesis in GO-derived OFs. **a** Representative images and **b** quantification of Nile red staining in differentiated OFs from normal and GO patients with naïve or hPMSCs^PRL-1^ co-culture for 24 h. **c** The mRNA expression of adipogenesis markers (e.g. *ADIPSIN*, *ADIPONECTIN*, *LPL*, *LEPTIN*, *PPAR****γ***, *FABP4*, *C/EBPα*, and *C/EBPβ*) by qRT-PCR. Significantly different values between the groups are indicated with marks (#*p* < 0.05 vs normal non-coculture (-); *p < 0.05 vs normal or GO-OF non-co-culture; **p < 0.05 vs hPMSCs)

The anti-adipogenic effects of hPMSCs^PRL-1^ have been reported previously [14]. Under the same conditions, differentiated OFs from the normal model and GO patients co-cultured with naïve and hPMSCs^PRL-1^ were stained using Nile red to detect lipid droplets (Fig. 4a). Compared with normal OFs, GO-derived OFs distinctly showed lipid accumulations. Quantification of the Nile red positive area in naïve or hPMSCs^PRL-1^ co-culture groups revealed lower values. Interestingly, the hPMSCs^PRL-1^ co-culture results were statistically significant compared to the naïve co-culture (Fig. 4b). As expected, in the hPMSC^PRL-1^ co-culture, mRNA expressions of adipogenesis markers (e.g., *ADIPSIN*, *ADIPONECTIN*, *LPL*, *LEPTIN*, *PPAR****γ***, *FABP4*, *C/EBPα*, and *C/EBPβ*) were remarkably reduced compared to the naïve co-culture (Fig. 4c). In general, the symptom of de novo adipogenesis in GO is accompanied by inflammation [16]. Compared with normal OFs, GO-derived OFs induce inflammatory cytokines (e.g., *ICAM-1*, *IL-1β*, *IL-6*, *TNF-α*, *TGFβ1*, and *TGFβ2*). Interestingly, the hPMSCs^PRL-1^ co-culture group exhibited reduced inflammatory-related factors, except for *IL-1β* and *TGFβ2*, compared with the hPMSCs group (Supplementary Fig. S2). These results suggest that hPMSCs^PRL-1^ suppress adipogenesis and inflammation in differentiated OFs of GO patients.

### Enhanced hPMSCs co-culture regulates TSHR-SREBP2-HMGCR signaling in GO-derived OFs

Adipogenesis and adipocyte lipogenesis are associated with sterol regulatory element-binding protein 2 (SREBP2) [17]. SREBP2 is expressed in both the cytoplasm and nucleus. However, the mature form of SREBP2 is actively translocated into the nucleus by stimulation of TSH through the TSHR. This results in the activation of 3-hydroxy-3-methyl-glutaryl-coenzyme a reductase (HMGCR) expression [18]. Naïve and hPMSCs^PRL-1^ co-cultures revealed that mRNA expressions of *TSHR*, *SREBP2*, and *HMGCR* levels were downregulated. Interestingly, the hPMSCs^PRL-1^ co-culture remarkably reduced *SREBP2* and *HMGCR* expressions. Active SREBP2 and HMGCR protein expression induced the same results. These data suggest that hPMSCs^PRL-1^ may regulate the TSHR-SREBP2-HMGCR signaling pathway in differentiated OFs of GO patients.

## Discussion

In previous work, they found that genetic immunity exhibits pathological features of GO by injecting human TSHR A-subunit plasmid and by remodeling of orbit tissue [10, 11]. In these studies, orbital pathology was determined by interstitial inflammation of EOMs with CD3+ T cells and F4/80+ macrophages, fibrosis, and adipogenesis. Furthermore, in vivo magnetic resonance imaging (MRI) scans of the orbital section of mice provided obvious and quantitative evidence of orbital muscle hypertrophy along with eye protrusion (proptosis) [10]. MRI can be applied to diagnose GO as well as evaluate the response to GO treatment [19, 20]. GO animal models also showed IL-10, IL-6, and TNF-α cytokine responses to activated T cells in the study [11].

There are three kinds of TSHR monoclonal antibody (stimulating, blocking, and cleavage antibodies) that differ in the functional capabilities in GD patients [21]. The M22, a human monoclonal antibody (mAb) to the TSHR, is widely used and is a high affinity stimulating antibody; as such, it is considered to be an international standard [22]. Stimulating antibodies reportedly induce thyrocyte cell survival and proliferation via cAMP/PKA/CREB and Akt/mTOR/S6k signaling. In contrast, cleavage antibodies would result in apoptosis via reactive oxygen species induction and nuclear factor kappa-B cell (NF-κB) activation. The balance between negative and positive regulation may be important for thyrocyte homeostasis in GD [21].

**Fig. 5:**
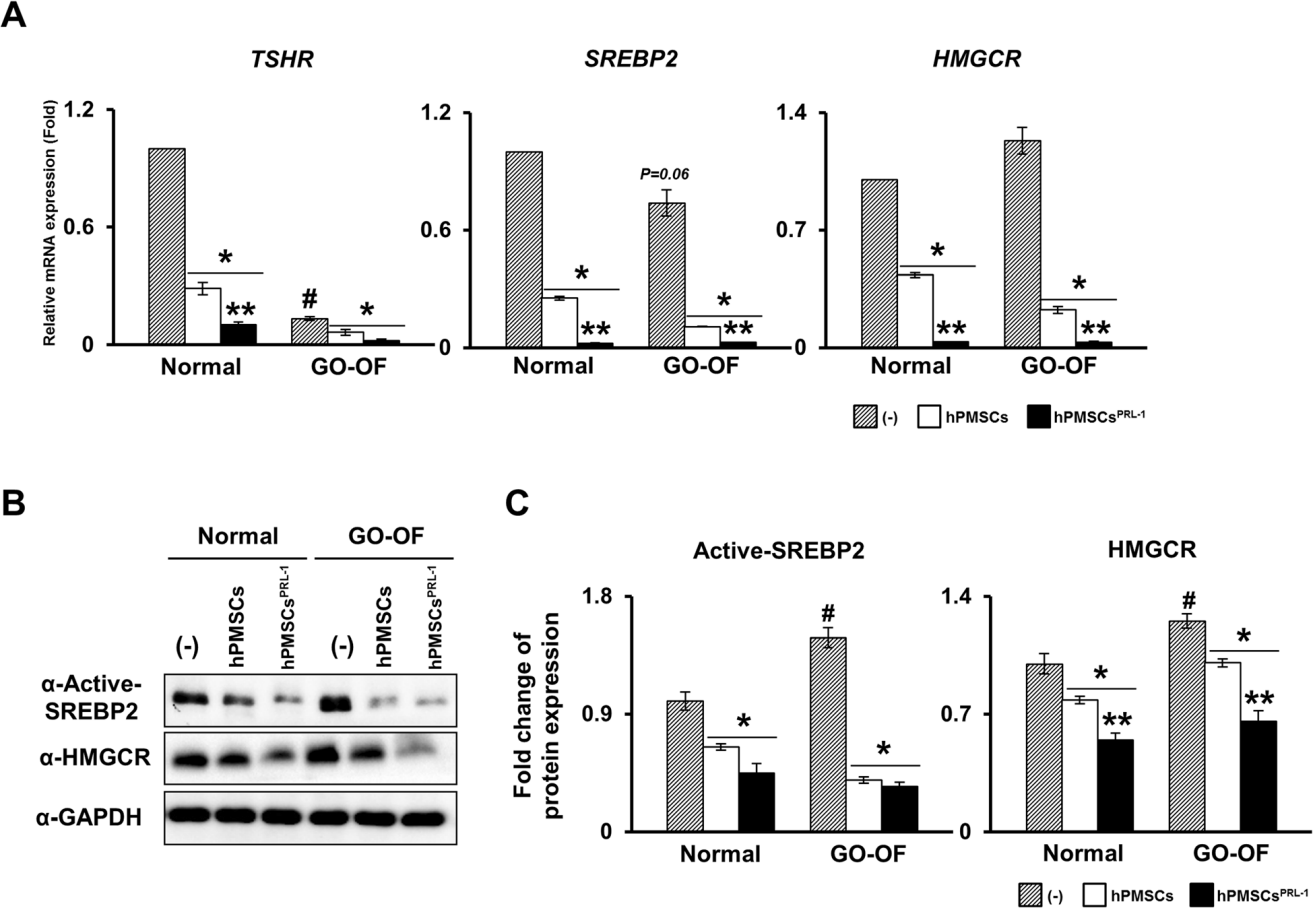
hPMSCs^PRL-1^co-culture regulate TSHR-SREBP2-HMGCR signaling pathway. The **a** mRNA, **b** protein expression, and **c** their quantifications of TSHR, active-SREBP2, and HMGCR in differentiated OFs from normal and GO patients with naïve or hPMSCs^PRL-1^ co-culture for 24 h. Significantly different values between the groups are indicated with marks (#*p* < 0.05 vs normal non-coculture (-); *p < 0.05 vs normal or GO-OF non-co-culture; **p < 0.05 vs hPMSCs)

**Fig. 6:**
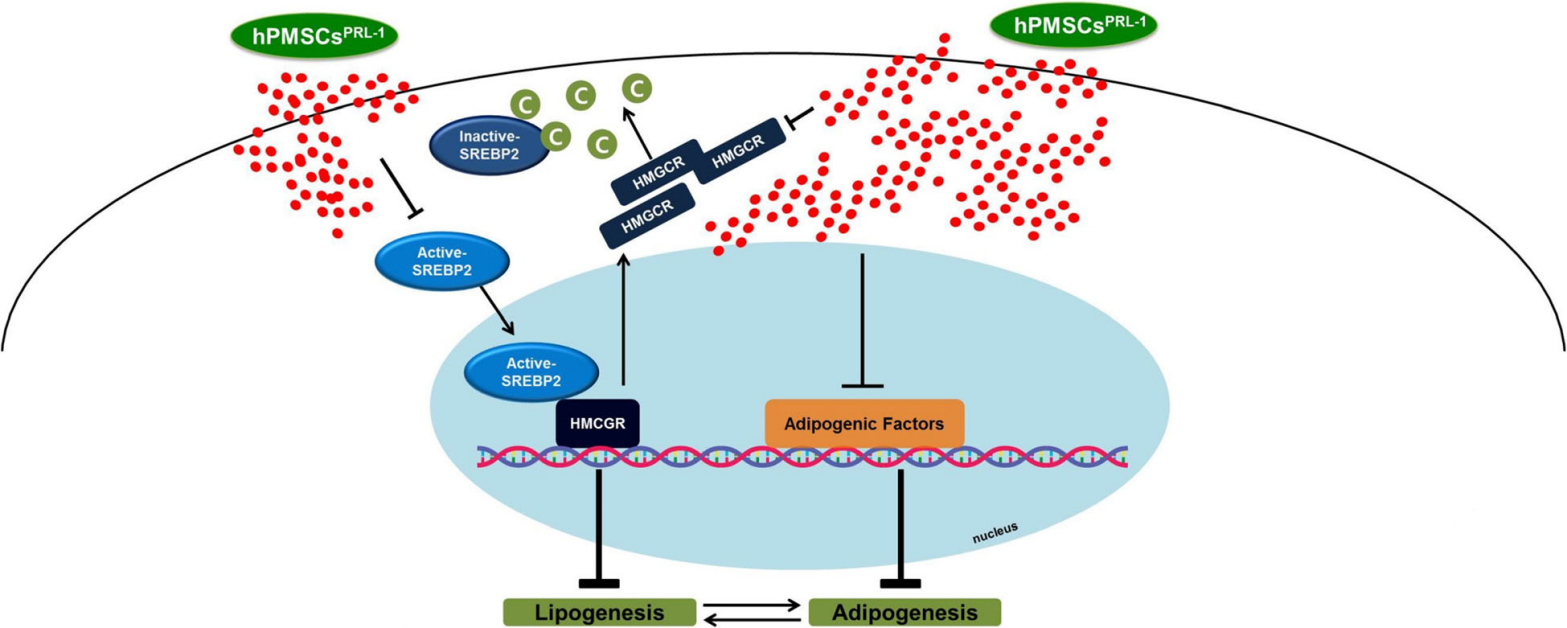
Proposed pathway of inhibition of hPMSCs^PRL-1^ for adipogenesis in GO

Non-specific anti-inflammatory drugs are commonly used in moderate to severe, active thyroid-associated orbitopathy (TAO). However, the effect and response of systemic corticosteroids injections vary from case to case [23]. Because glucocorticoid is associated with the risk of side effects, various treatments have been advanced for specific targets. Teprotumumab (a repurposed IGF-1R inhibitor), rituximab (anti-CD20), B cell depleting agents, and tocilizumab may be other candidates for recent TAO [20, 23]. In this study, we demonstrated that hPMSCs^PRL-1^ strongly downregulates the SREBP2-HMGCR signaling pathway in GO fibroblasts (Fig. 5). A number of transcriptional factors and enzymes, such as PPAR**γ**, SREBP2, and HMGCR, are involved in adipogenesis. SREBP2 protein is considered to be a key factor during cholesterol biosynthesis [24, 25]. In cholesterol synthesis, SREBP2 translocates into the nucleus leading to mRNA activation of HMGCR [25]. HMGCR-deficient adipocytes lead to a loss of lipid accumulation and increased apoptosis [26]. Furthermore, PPAR**γ** agonists have been shown to increase HMGCR induction [26, 27]. Figure 6 shows our model as to the mechanism of action of hPMSCs^PRL-1^ cells on inhibition of adipogenesis by suppression of the adipogenic factors in the mouse GO model.

In conclusion, studies of various pathologies have been continuously described; however, there was lack of research on the definite treatment effects of hPMSCs in GO. This is the initial research on the treatment effects of functionally enhanced placenta-derived mesenchymal stem cells on GO models. Based on our findings, we propose that hPMSCs^PRL-1^ have a robust anti-adipogenesis property, through the immunomodulation effects. Therefore, these results also demonstrate the potential of hPMSCs^PRL-1^ as a new cell therapy for GO disease.

## Supplementary Information

**Additional file 1: Supplementary Figure S1.** hPMSCs^PRL-1^ attenuated inflammatory related proteins level in GO animal model. The resultant changes in (A*) Icam-1*, *Il-1β*, *Il-6*, *Tnf-α* and *Tgfβ2* mRNA expression (*n* = 5/each group) and (B) Icam-1, Tgfβ2 and Tsh-receptor proteins expression of GO mice orbital tissues (all groups *n* = 3, except hPMSCs *n* = 5) were examined. Expression levels were normalized to (A) 18 s rRNA or (B) α-tubulin, and the quantified values of target proteins expression are also presented (right panel). Significantly different values between groups are indicated with asterisk (*P < 0.05, **P < 0.005, ***P < 0.001 vs age-matched sham; #*p* < 0.05).

**Additional file 2: Supplementary Figure S2.** hPMSCs^PRL-1^ co-culture attenuate inflammatory response in GO-derived OFs mRNA expression of inflammatory genes (e.g. *ICAM-1*, *IL-1β*, *IL-6*, *TNF-α*, *TGF-β1*, and *TGF-β2*) in differentiated OFs from normal and GO patients with naïve or hPMSCs^PRL-1^ co-culture for 24 h using qRT-PCR. Significantly different values between the groups are indicated with marks (#*p* < 0.05 vs Normal non-coculture (-); *p < 0.05 vs Normal or GO-OF non-coculture; **p < 0.05 vs hPMSCs).

## Data Availability

All data and materials are available upon request.

## Abbreviations

GO: Graves’ ophthalmopathy
GD: Graves’ disease
TAO: Thyroid-associated orbitopathy
OFs: Orbital fibroblasts
hPMSCs: Human placental mesenchymal stem cells
TSH: Thyroid-stimulating hormone
TSHR: Thyroid-stimulating hormone receptor
EOMs: Extraocular muscles
