# Supplementary figures and images for: PRL-1 overexpressed placenta-derived mesenchymal stem cells suppress adipogenesis in Graves’ ophthalmopathy through SREBP2/HMGCR pathway
Source: Stem Cell Res Ther. 2021 May 29;12:304. doi: 10.1186/s13287-021-02337-2 (PMC8164285; doi:10.1186/s13287-021-02337-2)

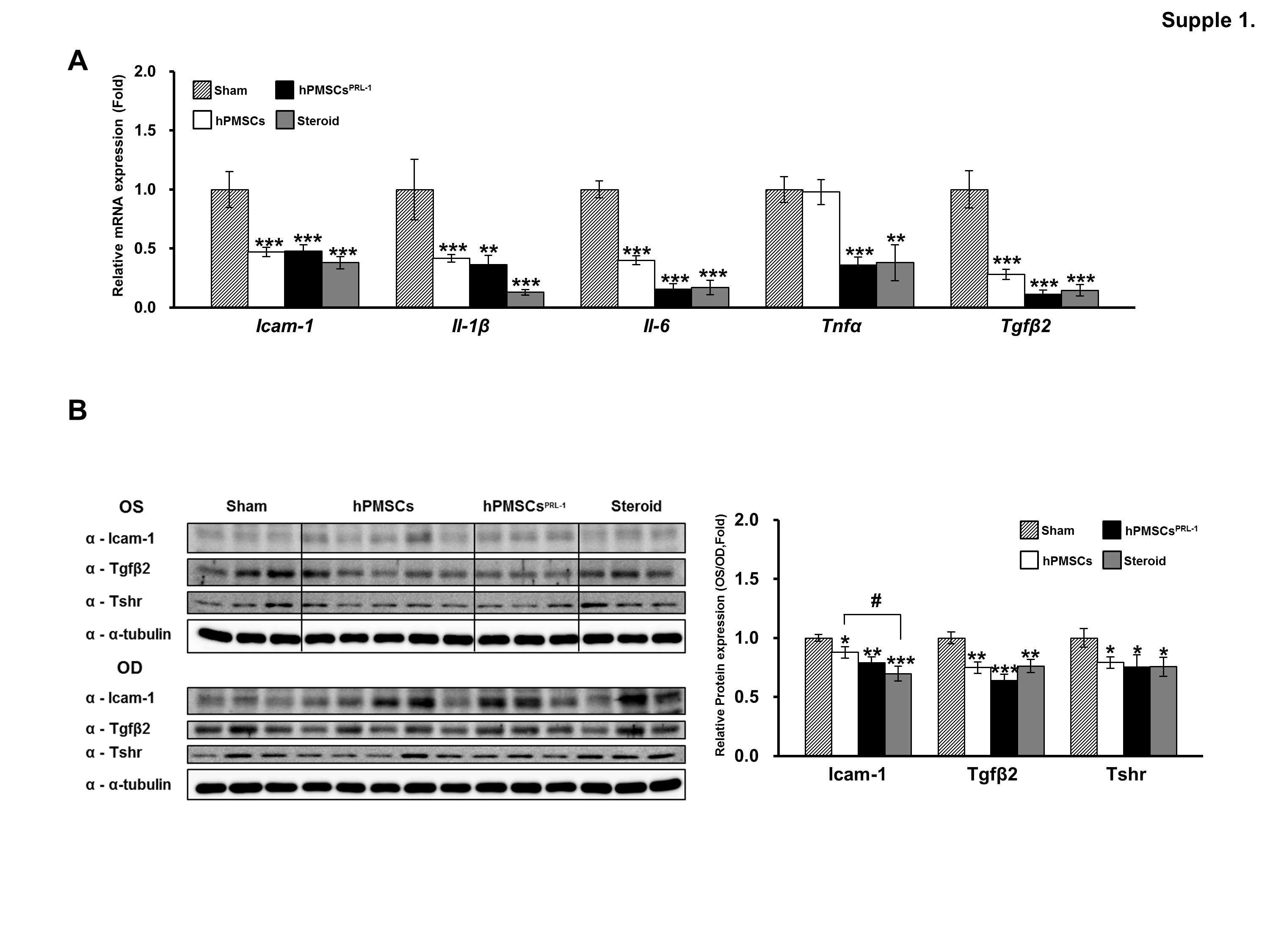

Supplement: Supplementary file 1 — Additional file 1: Supplementary Figure S1. hPMSCsPRL-1 attenuated inflammatory related proteins level in GO animal model. The resultant changes in (A) Icam-1, Il-1β, Il-6, Tnf-α and Tgfβ2 mRNA expression (n = 5/each group) and (B) Icam-1, Tgfβ2 and Tsh-receptor proteins expression of GO mice orbital tissues (all groups n = 3, except hPMSCs n = 5) were examined. Expression levels were normalized to (A) 18 s rRNA or (B) α-tubulin, and the quantified values of target proteins expression are also presented (right panel). Significantly different values between groups are indicated with asterisk (*P < 0.05, **P < 0.005, ***P < 0.001 vs age-matched sham; #p < 0.05). [file 13287_2021_2337_MOESM1_ESM.tif]

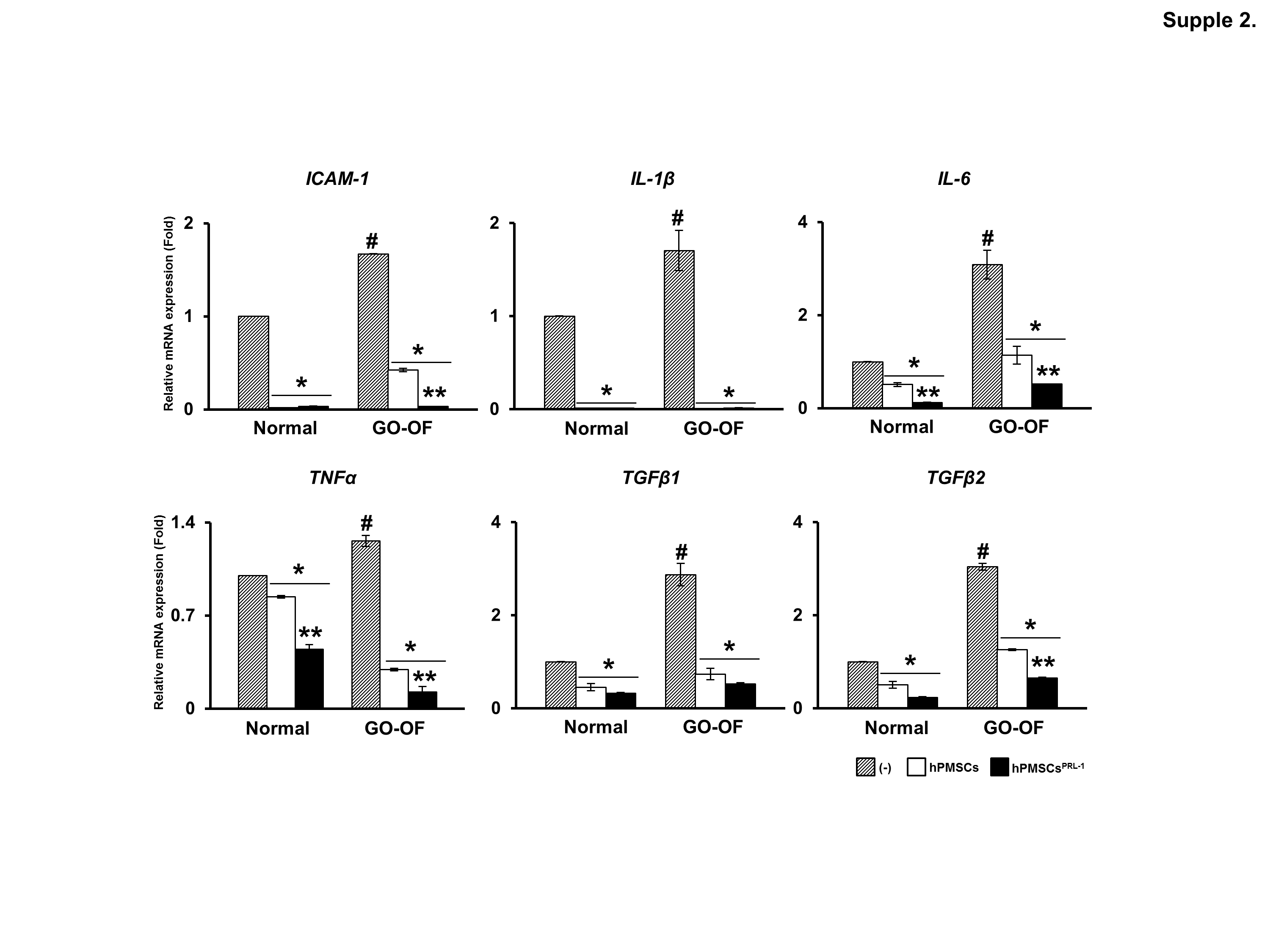

Supplement: Supplementary file 2 — Additional file 2: Supplementary Figure S2. hPMSCsPRL-1 co-culture attenuate inflammatory response in GO-derived OFs mRNA expression of inflammatory genes (e.g. ICAM-1, IL-1β, IL-6, TNF-α, TGF-β1, and TGF-β2) in differentiated OFs from normal and GO patients with naïve or hPMSCsPRL-1 co-culture for 24 h using qRT-PCR. Significantly different values between the groups are indicated with marks (#p < 0.05 vs Normal non-coculture (-); *p < 0.05 vs Normal or GO-OF non-coculture; **p < 0.05 vs hPMSCs). [file 13287_2021_2337_MOESM2_ESM.tif]
